# Single-Stage Bilateral Saucerization for Discoid Lateral Meniscus Tears in a Pediatric Patient: A Case Report

**DOI:** 10.7759/cureus.111001

**Published:** 2026-06-16

**Authors:** Josh T Morrow, Jason Z Amaral, Indranil V Kushare

**Affiliations:** 1 Department of Orthopedic Surgery, Texas Children's Hospital, Baylor College of Medicine, Houston, USA; 2 Department of Orthopaedic Surgery, University of Arizona College of Medicine – Phoenix, Phoenix, USA

**Keywords:** arthroscopy, discoid lateral meniscus, meniscus tear, pediatric orthopedics, single-stage saucerization

## Abstract

A 12-year-old boy presented with bilateral knee pain, and magnetic resonance imaging (MRI) revealed bilateral complete discoid lateral meniscus (DLM) tears. He underwent single-stage bilateral saucerization and was able to return to sports. Within four years, he sustained new tears in both knees during sports participation and underwent repeat arthroscopy. At the last follow-up, he had returned to unrestricted activity with improved symptoms. Single-stage bilateral saucerization may be a feasible and well-tolerated option for select pediatric patients with DLM tears, potentially minimizing cumulative recovery time. Further research is warranted to evaluate long-term outcomes and guide patient selection.

## Introduction

Discoid lateral meniscus (DLM) is the most common anatomical variant of the meniscus, characterized by an abnormally thickened and widened morphology that alters normal biomechanics and increases susceptibility to tearing. The estimated prevalence of DLM in pediatric and adolescent patients ranges from 0.4% to 20%, with both knees affected in up to 95% of cases [1,2].

Symptomatic DLM tears may present with mechanical symptoms such as snapping, locking, or joint line pain [3,4]. While conservative management may be attempted initially, persistent or activity-limiting symptoms often necessitate arthroscopic saucerization with or without meniscal repair [4-6]. In patients with bilateral symptomatic DLM, a two-stage surgical approach is typically performed to avoid the challenges of concurrent bilateral recovery [7,8]. However, there is limited literature describing single-stage bilateral treatment in pediatric patients.

This report presents a case of a 12-year-old boy with bilateral complete DLM tears treated with single-stage bilateral arthroscopic saucerization, with over four years of follow-up documenting early recovery and subsequent new tears in both knees.

## Case presentation

A 12-year-old boy with no significant medical or family history presented with right lateral knee pain and a popping sensation for 18 months following a skateboard injury. Physical examination demonstrated lateral joint line tenderness, swelling, and positive McMurray and Thessaly tests. Radiographs were unremarkable. Given the chronic presentation and absence of severe functional limitation, an initial trial of physical therapy was pursued for symptom control and functional improvement, with improvement in symptoms after six weeks.

Two years later, he sustained a painful pop in his left knee while on a trampoline. Examination demonstrated knee effusion, tenderness over the lateral joint line, and a positive McMurray test. MRI of the left knee demonstrated a complete DLM with a vertical longitudinal tear involving the posterior body and posterior horn (Figure 1). Given his persistent symptoms in the contralateral knee, an MRI of the right knee was also obtained, showing a complete DLM with degenerative intrasubstance signal abnormality and suspected nondisplaced posterior horn tear extending to the superior articular surface (Figure 2).

**Figure 1:**
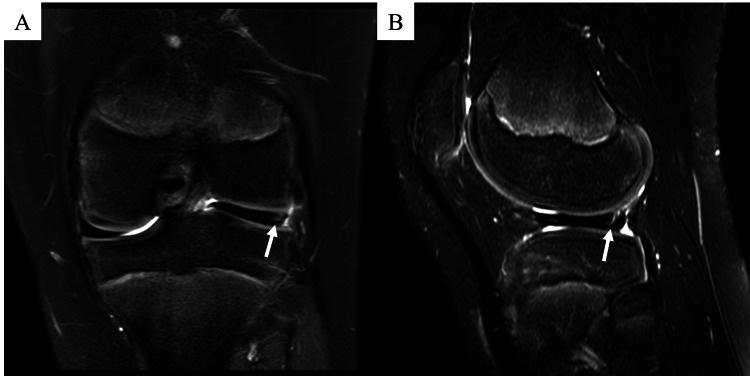
Coronal (a) and sagittal (b) T2 fat-suppressed MRI images of the left knee obtained three weeks after the initial injury, demonstrating a complete discoid lateral meniscus with a vertical longitudinal tear involving the posterior body and posterior horn.

**Figure 2:**
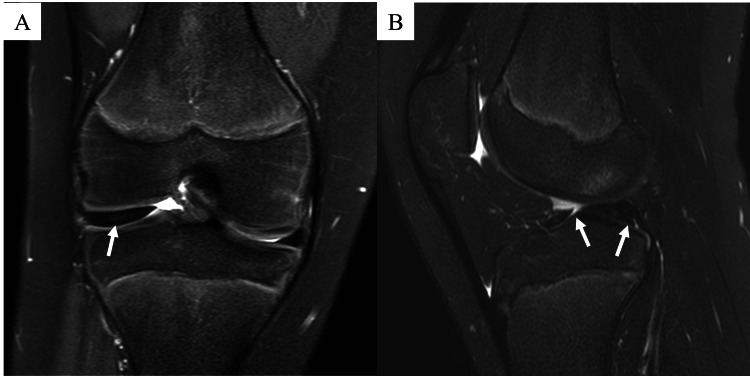
Coronal (a) and sagittal (b) T2 fat-suppressed MRI images of the right knee obtained three weeks after the injury, demonstrating a complete discoid lateral meniscus with degenerative intrasubstance signal abnormality and a suspected nondisplaced posterior horn tear extending to the superior articular surface.

Surgical intervention was recommended. After a thorough discussion of risks and benefits, the patient and family expressed a strong preference for a single-stage procedure due to concerns about repeat anesthesia and recovery burden. The decision was made to proceed with single-stage bilateral arthroscopic saucerization.

During surgery, both knees demonstrated complete discoid lateral menisci. On the left, a vertical body tear in the white zone was identified, and on the right, a posterior horn tear was identified. The remaining intra-articular structures were normal bilaterally on diagnostic arthroscopy. Bilateral saucerization was performed using a combination of biters and shavers. The remaining meniscal rims of around 9-10 mm were probed and found to be stable.

He was discharged home with instructions to bear weight as tolerated on both legs using crutches, follow up with physical therapy, and refrain from sports for at least six months.

At one-month follow-up, he reported no pain or mechanical symptoms and was cleared to return to non-impact activities. At three months, he presented with medial patellar tenderness in the left knee after playing basketball, but the right knee remained asymptomatic. Radiographs showed bilateral Osgood-Schlatter disease. Patellar straps were prescribed, and the patient was advised to limit high-impact activities. At the six-month postoperative visit, the patient reported resolution of left knee pain, with the right knee remaining asymptomatic. He continued physical therapy. At 11 months, he remained pain-free and with unrestricted activity.

Follow-up: left knee

At 21 months postoperatively, the patient developed pain, swelling, and popping in the left knee after landing awkwardly while playing basketball. Rest and physical therapy were recommended. At 33 months, repeat MRI identified a new radial tear involving the body and posterior horn of the left DLM, along with associated loose bodies and an osteochondral defect of the lateral femoral condyle with full-thickness chondral loss (Figure 3). At 36 months, he underwent repeat arthroscopy, which confirmed these findings. The procedure included meniscal debridement, chondroplasty, removal of loose bodies, and harvesting of autologous cartilage for possible future matrix-induced autologous chondrocyte implantation. The postoperative course was uneventful, and he remained symptom-free at 44 months from the index procedure (8 months after repeat arthroscopy).

**Figure 3:**
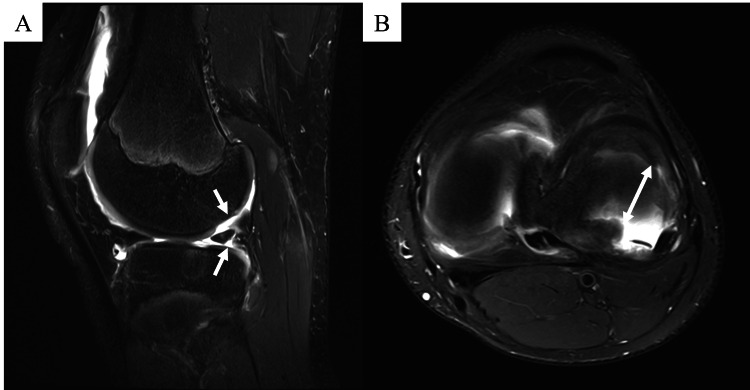
Sagittal (a) and axial (b) T2 fat-suppressed MRI images of the left knee obtained three years after the initial surgery demonstrate a diastatic radial tear involving the body/posterior horn of the lateral meniscus, with an osteochondral defect of the lateral femoral condyle and associated full-thickness chondral loss.

Follow-up: right knee

At 41 months postoperatively, the patient presented with right lateral knee pain, swelling, and mechanical symptoms after throwing a football. Physical examination demonstrated a positive McMurray test. Repeat MRI showed a new tear involving the anterior and posterior horns of the right DLM (Figure 4). He underwent repeat arthroscopy, which confirmed the MRI findings, and meniscal debridement was performed. The postoperative course was uneventful, and he remained symptom-free at 47 months after the index procedure (6 months after repeat arthroscopy).

**Figure 4:**
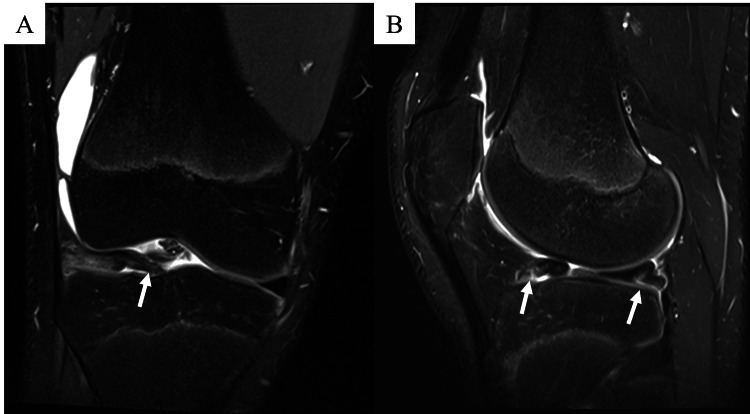
Coronal (a) and sagittal (b) T2 fat-suppressed MRI images of the right knee obtained four years after the initial surgery, demonstrating a complex tear involving the lateral meniscus.

## Discussion

In pediatric patients with bilateral symptomatic DLM tears, a staged surgical approach is typically performed to minimize the potential burden of simultaneous functional limitations in both lower extremities. This case illustrates that single-stage bilateral arthroscopic saucerization can be technically feasible and well tolerated in a skeletally immature patient, with early return to function and no postoperative complications.

In the present case, the patient returned to sports at 11 months postoperatively following single-stage bilateral saucerization. Although single-stage approaches for bilateral DLM are rarely reported, similar strategies have been described in pediatric patients with bilateral DLMs [9,10]. One report described a 10-year-old boy who underwent simultaneous saucerization followed by 4 weeks of non-weight bearing and progressive rehabilitation, ultimately returning to sport by 6 months [9]. Another described a 14-year-old girl who returned to competitive athletics within 4 months after a similar surgical and rehabilitation course [10]. While these cases involved the medial meniscus, which may differ biomechanically from the lateral variant, their favorable outcomes suggest that single-stage bilateral arthroscopy can be well tolerated in select pediatric patients [11]. Further studies are needed to evaluate the safety, efficacy, and long-term outcomes of this approach in patients with bilateral DLM tears.

The patient later sustained symptomatic new tears in both knees, first the left at 36 months and then the right at 41 months, each associated with acute sports-related trauma. These events are consistent with prior studies reporting high retear rates following DLM saucerization, with rates approaching 60% by eight years [12,13]. Additionally, younger age and open physes have been identified as risk factors for retear [12]. In this case, the tears occurred at new sites and may reflect the patient’s skeletal immaturity and participation in high-impact activities rather than the single-stage approach. These findings underscore the importance of individualized patient selection and preoperative counseling [14]. Future studies should evaluate retear risk following single-stage bilateral arthroscopy to help identify optimal candidates for this approach.

Single-stage bilateral saucerization may offer practical benefits in select pediatric patients, such as reduced anesthesia exposure, fewer disruptions to school and rehabilitation, and potentially lower overall healthcare utilization [15]. However, these potential advantages must be weighed against the potential demands of bilateral recovery and the risk of early overloading. Future studies should evaluate long-term outcomes following single-stage arthroscopy to inform risk-benefit discussions and guide shared decision-making with patients and families.

## Conclusions

Single-stage bilateral saucerization may be a technically feasible and well-tolerated option for select pediatric patients with DLM tears and may reduce cumulative recovery time. While retear risk remains a concern in cases of DLM, particularly among skeletally immature and highly active patients, this approach may be appropriate when individualized factors support a one-stage strategy. Larger studies are needed to guide patient selection and clarify long-term outcomes.
